# Investigation of gastric cancers in nude mice using X-ray in-line phase contrast imaging

**DOI:** 10.1186/1475-925X-13-101

**Published:** 2014-07-24

**Authors:** Qiang Tao, Shuqian Luo

**Affiliations:** 1 Biomedical Engineering Institute, Capital Medical University, Beijing 100069, China

**Keywords:** X-ray in-line phase contrast imaging, X-ray absorption imaging, Gastric cancer, Principal component analysis, Support vector machine

## Abstract

**Background:**

This paper is to report the new imaging of gastric cancers without the use of imaging agents. Both gastric normal regions and gastric cancer regions can be distinguished by using the principal component analysis (PCA) based on the gray level co-occurrence matrix (GLCM).

**Methods:**

Human gastric cancer BGC823 cells were implanted into the stomachs of nude mice. Then, 3, 5, 7, 9 or 11 days after cancer cells implantation, the nude mice were sacrificed and their stomachs were removed. X-ray in-line phase contrast imaging (XILPCI), an X-ray phase contrast imaging method, has greater soft tissue contrast than traditional absorption radiography and generates higher-resolution images. The gastric specimens were imaged by an XILPCIs’ charge coupled device (CCD) of 9 μm image resolution. The PCA of the projective images’ region of interests (ROIs) based on GLCM were extracted to discriminate gastric normal regions and gastric cancer regions. Different stages of gastric cancers were classified by using support vector machines (SVMs).

**Results:**

The X-ray in-line phase contrast images of nude mice gastric specimens clearly show the gastric architectures and the details of the early gastric cancers. The phase contrast computed tomography (CT) images of nude mice gastric cancer specimens are better than the traditional absorption CT images without the use of imaging agents. The results of the PCA of the texture parameters based on GLCM of normal regions is (F_1_ + F_2_) > 8.5, but those of cancer regions is (F_1_ + F_2_) < 8.5. The classification accuracy is 83.3% that classifying gastric specimens into different stages using SVMs.

**Conclusions:**

This is a very preliminary feasibility study. With further researches, XILPCI could become a noninvasive method for future the early detection of gastric cancers or medical researches.

## Background

Cancer is the world’s second leading cause of morbidity. Gastric cancer is one of the most frequent causes of cancer-related death in Asia [1]. Early detection and early treatment of gastric cancers are still the focus of cancer prevention and treatment. X-ray traditional imaging of the human skeleton provides high-resolution images, but that of the human abdominal organs is very poor. In recent years, a new imaging method, the X-ray in-line phase contrast imaging (XILPCI) has emerged. This imaging method is mainly based on the X-ray phase change factor after an X-ray passes through objects. XILPCI of soft tissues provides micrometer spatial resolution.

Early cancer detection mainly depends on radiographic imaging. The current examination methods of stomachs mainly consist of CT [2-5], magnetic resonance imaging (MRI) [4,6], endoscopy [7,8] and gas-barium double contrast X-ray gastrointestinalgraphy [9,10]. The image resolution of these devices is on the millimeter-scale. The image resolution that can be achieved by X-ray phase contrast imaging (XPCI) is on the micron-scale. The X-ray phase shift is approximately 1000 times greater than the change in absorption. Currently, there are a number of international research teams proposing various contrast imaging methods. The most commonly used approaches build XPCI systems include X-ray interferometer [11-13], diffraction enhanced imaging [14-16], XILPCI [17,18] and X-ray grating interferometer [19].

Spatial resolution expresses the power to resolve fine structures. Density resolution (i.e., contrast resolution) expresses subtle density differences. Currently, the spatial resolution of micro-CT has can reach 2 μm [20] and micro-CT can distinguish a tissue density difference of 0.01 g/cm^3^[21], but the image resolution of micro-CT is still at millimeter-scale without the use of imaging agents. MRI provides good contrast resolution and spatial resolution of soft tissues, but MRI image resolution is only at the millimeter-scale. The spatial resolution is limited by the magnetic strength of the MRI and is difficult to further increase.

Currently, the early detection of gastric cancers is mainly dependent on endoscopy and it is confirmed by a biopsy. The image resolution of endoscopy is about 0.56 mm [22]. Patients felt pain in the process of examination, and there was the risk of perforation and bleeding.

Gas-barium double contrast X-ray gastrointestinalgraphy is a common clinical tool to assess gastrointestinal conditions. CO_2_-barium is most commonly used, because of higher security and lower price. Before the examination, some aerogenic powders are taken orally by patients. Reactions between the dry powders won’t take place until they encounter water. The gastrointestinal tract will be expanded by the produced CO_2_ gas. After a few minutes, the patients take orally barium. The CO_2_-barium double contrast X-ray imaging can not only provide optimal visualization of mucosal abnormalities, but also evaluate the intestinal peristaltic function [9]. However, the method is prohibited to use, when a patient is suspected of gastrointestinal perforation or complete obstruction. The image resolution is at the millimeter-scale. In the process of examination, patients must receive multiple X-ray irradiations. It is slow to empty barium in the body of the patient after examination.

XILPCI is combined with CT, which provides images based on phase tomography. Phase contrast CT is also known as diffraction CT [23] and is a potentially useful imaging method for soft tissues without the use of imaging agents. The XILPCI image resolution of soft tissues can reach 0.74 μm without the use of imaging agents. It increases the accuracy of the detection and can be used to observe early cancer lesions.

## Methods

### Setup and specimens

Nude mice do not possess normal thymuses and only have thymus remnants or abnormal thymus epithelial, which cannot produce T cells by normal thymus epithelial division. The lymph nodes and spleen lymphocytes of nude mice are very small, so nude mice are animals possessing fewer lymphocytes and nude mice also display skin and hair atrophy and follicular keratosis. In general, nude mice are considered to be the closest human genetic model among laboratory animals for studying human diseases. A variety of human cancers are generally able to survive in nude mice. Due to their immune deficiency, nude mice do not reject tissues from other animals. Therefore, they can be used as recipients for the transplantation of malignant human cancers.

In the pre-test process, both to simulate the physiological conditions of the human stomachs and to obtain clear images, we found that the image was very clear to meet the requirements of our experiments, when we used the gastric specimens cleaned out of food residue and filled with air. Therefore, we decided to use the air-filled gastric specimens for the remaining experiments.

The nude mice were female and weighing approximately 16 g in our experiments. A total of 36 nude mice were randomly divided into 6 groups for our experiments and each group is 6 nude mice. One group is nude mouse normal group, and the other 5 groups are nude mouse gastric cancer groups. The nude mice in nude mouse gastric cancer groups were anesthetized with an intraperitoneal injection of 0.72 mg (45 mg/kg) pentobarbital sodium. After it was anesthetized, each nude mouse received a transverse incision in its abdomen. The stomach was extruded, an incision was made and human gastric cancer BGC823 cells [24] were transplanted into the nude mouse stomach. Then, the wound was sutured. Each operation lasted about 10 minutes. After approximately one hour, the nude mouse awoke. After the implantation of gastric cancer cells, 2 days were allowed to pass to allow for possible animal immune responses. After 3, 5, 7, 9 or 11 days, the nude mice were sacrificed and the stomachs of the nude mice were removed. The stomachs were cleaned and filled in formalin. The esophagus and duodenum near the stomach were separately trussed by suture. The gastric specimens were fixed in a 10% formalin solution. The animal study was approved by the Experimental Animal Ethics Committee. The quality certification of animals is Sheng Chan Xv Ke (SCXK Beijing) 2005–0004.

### Principle of XILPCI

Synchrotron radiation [25] is electromagnetic radiation in which charged particles are accelerated to nearly the speed of light in a magnetic field by the Lorentz force when moving at variable speed along the track tangent direction. As a light source, its advantages are obvious: wide band, high collimation, high polarization, high purity, high brightness, narrow pulse and high coherence. Synchrotron radiation has a high degree of stability, high throughput and a micro-beam diameter.

**Figure 1:**
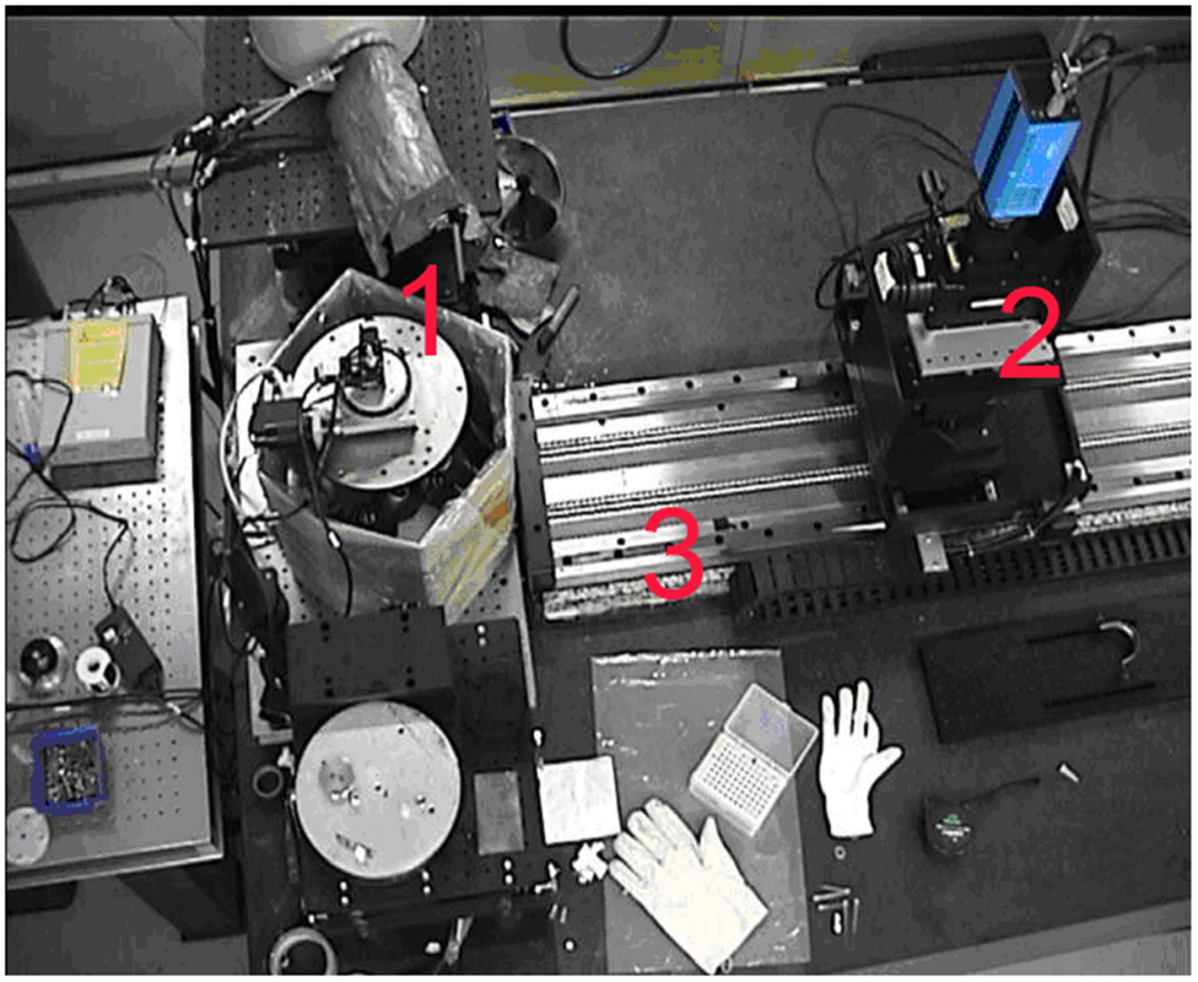
**The picture of BL13W1 beamline partial facility of SSRF.** 1. A multidimensional specimen table. The specimens are placed on the specimen table to rotate and specimens were obtained in different angles. 2. An X-ray CCD. It obtained specimens’ projective images with high-resolution. 3. The precise guide rail. It can control the exact distance from the CCD to the specimens.

The XILPCI experiments were performed using the BL13W1 beamline of the Shanghai Synchrotron Radiation Facility (SSRF) [26]. The BL13W1 beamline mainly produced 2-Dimensional images of biological tissues using XILPCI. The BL13W1 beamline partial facility of SSRF was depicted as Figure 1. XILPCI is also termed Fresnel diffraction [27,28] or coaxial phase contrast imaging. In 1995, A. Snigirev [29] used a synchrotron light source to obtain phase contrast images. The XILPCI method does not require temporal coherence of the light source. It can use multi-color light sources, therefore eliminating the need for the burdensome complexity of the monochrome system. The method can directly use micro-focus X-ray sources instead of synchrotron radiation sources. This advantage may make XILPCI suitable for clinical medicine in the future.

When an X-ray goes through a specimen, as in ordinary optical, the complex refractive index can be used to describe their characteristics. The refractive index n is a slightly smaller than the number of 1, can be written as:

(1) n=1-δ-iβ

The real component δ represents phase; and imaginary part β represents absorption term. δ is associated with physical phase shift section P; and β is associated with the linear absorption coefficient of the material μ. The relationship between them is as follows:

(2) $$\delta =\frac{\rho_{e}r_{e}\lambda^{2}}{2\pi },\beta =\frac{\mu \lambda }{4\pi }$$

(3) $$P=\frac{2\pi \delta }{\lambda },\mu =\frac{4\pi \beta }{\lambda }$$

λ is X-ray wavelength, ρ_e_ is the electron density in the material, r_e_ is a classical electron radius, and their size is determined by the electron density of objects’ interior structures.

When an X-ray goes through the object, its phase and amplitude change. Phase change is determined by the δ, and amplitude attenuation is determined by the β. In X-rays, for lighter elements (such as C, H, O, etc.) of the material, δ is 1000 times more than β, so the phase change quantity is much larger than the change quantity of X-ray absorption attenuation. When an X-ray wavelength is very short, for weak absorption materials, the small changes of density also can produce large phase shifts, thereby gaining high phase contrast. The spatial resolution of phase contrast imaging can reach micron-scale and a very fine microstructure of an object can be observed.

When uniform intervening light waves pass through an uneven surface object, they inevitably generate phase changes, namely waves’ distortion. If the distortion waves continue to spread to a certain distance, the distortion waves will interfere with the non-distortion waves. Thus, it can be concluded that obtaining phase contrast images requires a coherent light source and appropriate distances from the light source to the specimen and from the specimen to the detector.

### Steps of XILPCI

Specific experimental methods: the nude mice gastric specimens containing transplanted human gastric cancer BGC823 cells were taken out from formalin, wrapped with insulating materials and placed on the specimen table.

We found that the X-ray beam energy of 13 keV was suitable for imaging experiment requirements, through we repeatedly debugged X-ray energy. It will make images too light if energy is higher than 13 keV. It will make imaging exposure time increase if energy is lower than 13 keV. Images will become dark if exposure time is too short. When we increased exposure time, it took us longer time to shoot more than 1000 images for CT image reconstruction. Gastric specimens will be led to serious deformation if the shoot time is too long. So 13 keV is an optimal parameter of comprehensive factors. The distance from the light source to the specimen is the length from the SSRF X-ray source to the gastric specimen on the specimen table. The distance was 59.3 m. The detector was 85 cm distance from the specimen, with 9 μm image resolution and the exposure time of 35–45 ms. It took approximately 20 minutes to obtain XILPCI projective images by 0.1 degrees steps from the degree of 0 to 180 of a gastric specimen.

### Steps of X-ray traditional absorption imaging

Traditional absorption CT images of gastric cancer specimens were done by using SIEMENS Inveon Scanners and Inveon Acquisition Workplace with 1.5 Service Pack. Gastric cancer specimens were put on the sample table and experiment parameters were debugged in operation room in order to meet the experiment requirements. The minimum resolution of this equipment was 11 μm. The energy of the X-ray was 80 keV and 400 μA. Energy parameters have been the equipment’s maximum power. A gastric cancer specimen was scanned by rotating 360°. It needed 967 s to scan a gastric cancer specimen and reconstruct absorption CT images at the same time.

## GLCM method

We used the 9 gray level co-occurrence matrix (GLCM) texture characteristics of angular second moment (ASM), inertia, inverse difference moment (IDM), entropy, correlation, sum average (SA), difference average (DA), sum entropy (SE), and difference entropy (DE) [30]. The GLCM is defined as C_ij_.

Angular second moment formula:

(4) $$T_{1}=\sum_{i=0}^{K‒1}\sum_{j=0}^{K‒1}C_{\mathrm{ij}}^{2}$$

Inertia formula:

(5) $$T_{2}=\sum_{i=0}^{K‒1}\sum_{j=0}^{K‒1}{\left(i‒j\right)}^{2}C_{\mathrm{ij}}$$

Inverse difference moment formula:

(6) $$T_{3}=\sum_{i=0}^{K‒1}\sum_{j=0}^{K‒1}\frac{1}{1+{\left(i‒j\right)}^{2}}C_{\mathrm{ij}}$$

Entropy formula:

(7) $$T_{4}=‒\sum_{i=0}^{K‒1}\sum_{j=0}^{K‒1}C_{\mathrm{ij}}\log C_{\mathrm{ij}}$$

Marginal distribution derived from GLCM.

(8) $$c_{x}\left(i\right)=\sum_{j=0}^{K‒1}c_{\mathrm{ij}}$$

(9) $$c_{y}\left(j\right)=\sum_{i=0}^{K‒1}c_{\mathrm{ij}}$$

*μ*_*x*_, *μ*_*y*_, *σ*_*x*_, *σ*_*y*_ respectively represent the mean and standard deviation of the marginal distribution.

The specify grayscale and the probability sum of difference between i and j express as follows:

(10) $$c_{x+y}\left(k\right)=\sum_{i+j=k}c_{\mathrm{ij}}\;k=0,1,2,\cdots ,2K‒2$$

(11) $$c_{x‒y}\left(k\right)=\sum_{\left|i‒j\right|=k}c_{\mathrm{ij}}\;k=0,1,2,\cdots ,K‒1$$

Correlation formula:

(12) $$T_{5}=\frac{\sum_{i=0}^{K‒1}\sum_{j=0}^{K‒1}\left(\mathrm{ij}\right)C_{\mathrm{ij}}‒\mu_{x}\mu_{y}}{\sigma_{x}\sigma_{y}}$$

Sum average formula:

(13) $$T_{6}=\sum_{k=0}^{2K‒2}kC_{x+y}\left(k\right)$$

Difference average formula:

(14) $$T_{7}=\sum_{k=0}^{K‒1}kC_{x‒y}\left(k\right)$$

Sum entropy formula:

(15) $$T_{8}=‒\sum_{k=0}^{2K‒2}C_{x+y}\left(k\right)\log\left\{C_{x+y}\left(k\right)\right\}$$

Difference entropy formula:

(16) $$T_{9}=‒\sum_{k=0}^{K‒1}C_{x‒y}\left(k\right)\log\left\{C_{x‒y}\left(k\right)\right\}$$

The XILPCI projective image is 256 grayscale, so it is enough to use the GLCM method to analyze texture characteristics of the XILPCI projective image. Texture characteristics analysis was used to explore the image characteristics and the texture characteristics thresholds of the gastric cancer projective images.

### PCA of the projective images texture based on GLCM

Firstly, selection 20 ROIs in each XILPCI projective image, we calculated 9 texture characteristics of each ROI based on GLCM. The size of each ROI was 50 × 50 pixels. A 20 × 9 texture data matrix T is expressed as follows:

(17) $$T=\left[\begin{array}{cccc} T_{11} & T_{12} & \cdots & T_{19}\\ T_{21} & T_{22} & \cdots & T_{29}\\ \vdots & \vdots & \vdots & \vdots \\ T_{201} & T_{202} & \cdots & T_{209} \end{array}\right]$$

Secondly, we solved the correlation coefficient matrix R of the texture data matrix T.

(18) $$R=\left[\begin{array}{cccc} r_{11} & r_{12} & \cdots & r_{19}\\ r_{21} & r_{22} & \cdots & r_{29}\\ \vdots & \vdots & \vdots & \vdots \\ r_{91} & r_{92} & \cdots & r_{99} \end{array}\right]$$

Thirdly, the r_ij_(i,j = 1,2,3…9) was the correlation coefficient of the original variable T, and r_ij_ = r_ji_, r_ij_’s calculation formula is as follows:

(19) $$r_{\mathrm{ij}}=\frac{\sum_{k=1}^{9}\left(T_{\mathrm{ki}}-\bar{T_{i}}\right)\;\left(T_{\mathrm{kj}}-\bar{T_{j}}\right)}{\sqrt{\sum_{k=1}^{9}{\left(T_{\mathrm{ki}}-\bar{T_{i}}\right)}^{2}\sum_{k=1}^{9}{\left(T_{\mathrm{kj}}-\bar{T_{j}}\right)}^{2}}}$$

Because the each variable of the texture data matrix T in the dimension was not exactly the same, we must standardize the texture data matrix T before solving the correlation coefficient of texture data matrix T.

Fourthly, the eigenvalues denoted as [*λ*_1_, *λ*_2_, *λ*_3_, ⋯ *λ*_9_] of the correlation coefficient matrix R. The contribution rate (CR) of each principal component was calculated and their formulas are as follows:

(20) $$\mathrm{CR}=\frac{\lambda_{i}}{\sum_{k=1}^{9}\lambda_{k}}\left(i=1,2\cdots 9\right)$$

The cumulative contribution rate (CCR) of principal components was calculated and their formulas are as follows:

(21) $$\mathrm{CCR}=\frac{\sum_{k=1}^{i}\lambda_{k}}{\sum_{k=1}^{9}\lambda_{k}}\left(i=1,2\cdots 9\right)$$

Lastly, the eigenvector denoted as [*l*_*i*1_, *l*_*i*2_, *l*_*i*3_ ⋯ *l*_*i*9_] of *λ*_*i*_. The new component can be expressed as follows:

(22) $$F_{i}={\left[\begin{array}{c} \begin{array}{cc} \begin{array}{cc} l_{i1} & l_{i2} \end{array} & l_{i3}\;\cdots \;l_{i9} \end{array} \end{array},\;\right]}^{T}\times \left[\begin{array}{c} \begin{array}{ccc} T_{1} & T_{2} & T_{3}\;\cdots \;T_{9} \end{array} \end{array},\;\right]\left(i=1,2,3,\cdots 9\right)$$

If the CCR of F_i_ is greater than 80%, we use F_i_ as the principal component.

PCA was carried out on the texture parameters we acquired above. Subsequently, the means and standard deviations of the principal components were computed to discriminate the normal and cancer regions.

### Gastric specimens classification using SVMs

SVMs are considered as a supervised computer learning method [31]. SVMs avoid several problems associated with unsupervised clustering methods, such as hierarchical clustering and self-organizing maps. This method avoids the computational burden of explicitly representing the feature vectors. The acquired texture parameters of the ROIs for each of gastric specimens were used as eigenvector. SVMs are used to classify gastric specimens into different stages.

In k-fold cross-validation, 10-fold cross-validation is commonly used. So we used 10-fold cross-validation. Leave-one-out is the extreme case of k-fold cross-validation, and it is suitable for a small amount of samples. There are a lot of calculations if dealing with a large number of samples using leave-one-out cross-validation. In 10-fold cross-validation, the original sample is randomly partitioned into 10 subsamples. Of the 10 subsamples, a single subsample is retained as the validation data for testing the model, and the remaining 9 subsamples are used as training data. The cross-validation process is then repeated 10 times. The 10 results from the folds then can be averaged to produce a single estimation. The advantage of this method over repeated random sub-sampling is that all observations are used for both training and validation, and each observation is used for validation exactly once.

## Results

**Figure 2:**
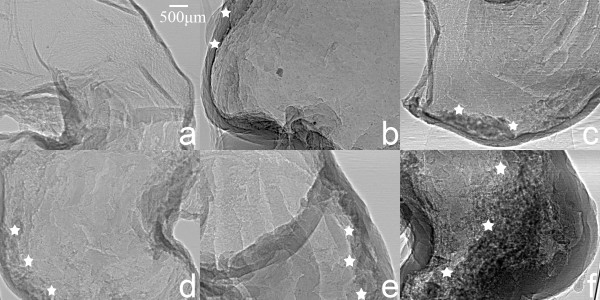
**XILPCI projective images of nude mice gastric specimens. (a)** An XILPCI projective image of a gastric normal specimen. **(b)** A 3-day-old specimen. It shows that the gastric walls are a little thickness and the surface of the stomach is uneven. **(c)** A 5-day-old specimen. It shows an obvious gastric cancer in the side of the stomach, but the cancer mass is small. **(d)** A 7-day-old specimen. It shows a larger region of a gastric cancer and the gastric thickening walls without smoothing. **(e)** A 9-day-old specimen. It shows that the gastric cancer regions continue to increase with more thickness gastric walls. **(f)** An 11-day-old specimen. It possesses the most thickness gastric walls. The gastric cancer has occupied more than half of the stomach. Star symbols represent suspected gastric cancer area.

**Figure 3:**
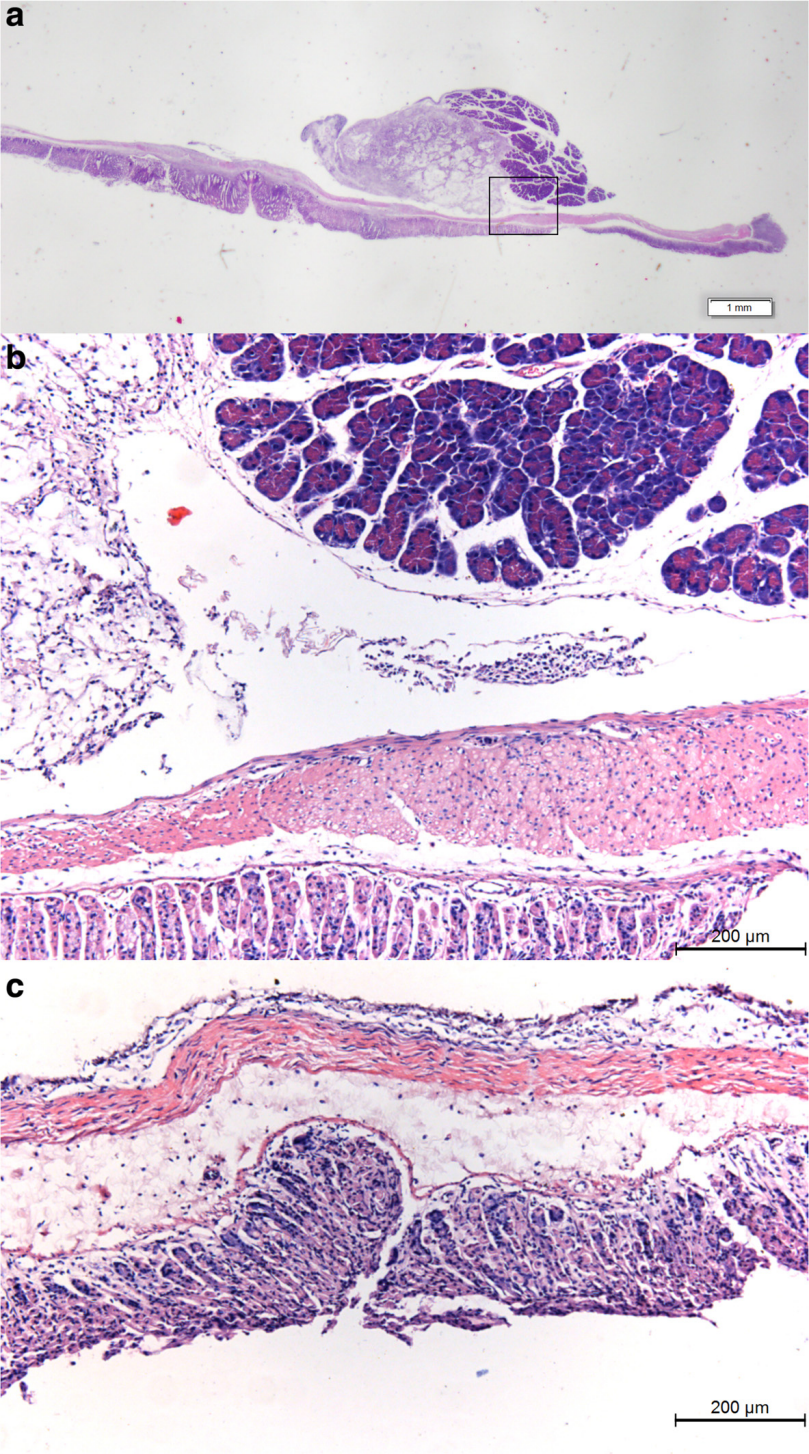
**Nude mice gastric HE pathological staining images. (a)** A 5-day-old gastric cancer; **(b)** A larger figure of black box section of Figure 3(a). It is visible malignant adenoma of irregular cell arrangement, huge nucleus, deep dyeing and cancer capsule intact; **(c)** A gastric normal specimen. It shows regular cell arrangement.

The resulting images of gastric experimental specimens are shown in Figure 2. We were able to observe changes at different days after gastric cancers implantation. Figure 2(a) presents a gastric normal specimen XILPCI image, demonstrating the characteristics of gastric normal tissues. The gastric normal tissues characteristics are ordered and regular and the gastric walls are smooth without any hyperplasia. The XILPCI image was more detailed than the X-ray traditional absorption image of a gastric normal specimen [32]. The marked regions of the Figure 2(b), (c), (d), (e) and (f) are gastric cancer tissues, which were identified by a clinical expert according to hematoxylin-eosin (HE) pathological staining images.The specimens were made for paraffin embedding, section 4 μm thick and HE pathological staining and observed under electron microscope (as Figure 3).

### Results of gastric cancer specimens CT

**Figure 4:**
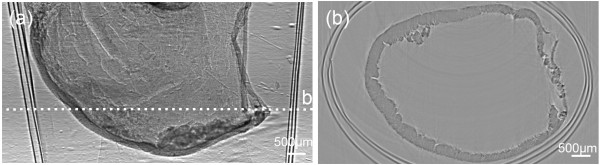
**The XILPCI CT image of the 5-day-old nude mouse gastric cancer specimen. (a)** The coronal image of nude mouse gastric cancer specimen. **(b)** The transverse image in dotted line position. It can obviously show that there are gray level changes in gastric different structures.

**Figure 5:**
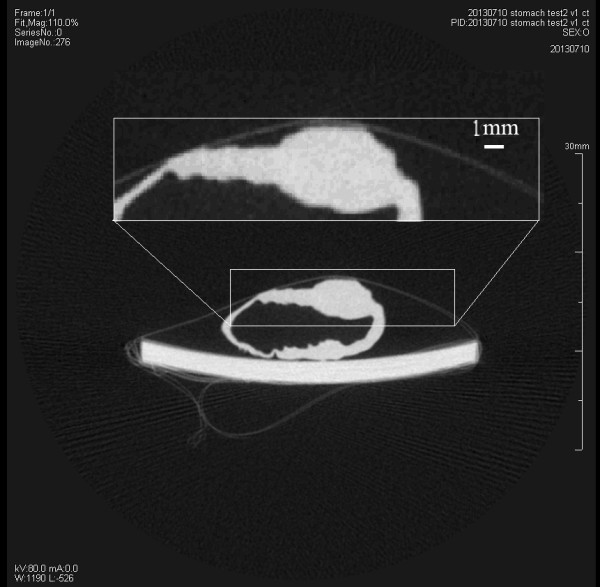
**The traditional absorption CT image of the 5-day-old nude mouse gastric cancer specimen.** There is no gray level change information and gastric wall is not clear.

We rebuilt the XILPCI 3-Dimensional slice using the filter back projective algorithm. The details can be observed inside the gastric tissue from tomography image in Figure 4.A traditional absorption CT image of a gastric cancer specimen illuminates that the whole gastric specimen looks like a white ring from Figure 5. It can be clearly observed that the gastric cancer region is protuberant thickness, but the details of the gastric wall are not clear.Phase contrast CT showed details better than absorption CT for gastric specimens according to Figures 4 and 5. The above conclusion was confirmed by a clinical image doctor.

### Results of GLCM of nude mice gastric XILPCI projective images

**Figure 6:**
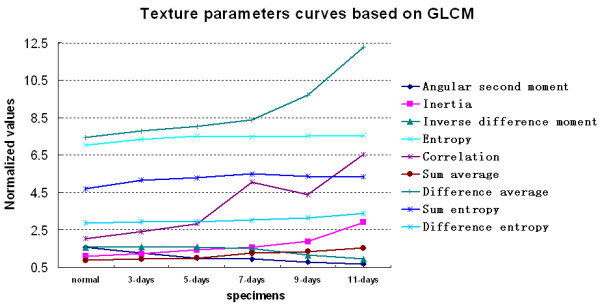
**A graph of the 9 texture parameters based on GLCM.** There are four texture parameters values (i.e. inertia, sum average, difference average and difference entropy) that significantly increase with the development of gastric cancer. Angular second moment and inertia represent the uniformity of the area. The data of angular second moment and inertia indicate that gastric cancer regions are not uniform and the gastric normal regions are relatively uniform. Entropy represents the regional regular texture, and the entropy value of regional structure texture is small. A gastric normal region has relatively regular texture, which is different from the gastric cancer region by entropy values.

We calculated the results of 9 texture parameters based on GLCM which are shown in Figure 6. Because of the presence of the slow development of early cancer in the individual vivo, it is not obvious differences among the texture parameters values of the normal, 3-days and 5-days gastric specimens. Because of the different development of gastric cancer in the individual vivo and the randomness of selection ROIs, these uncertainties make 7-days gastric specimen significantly changed in the correlation values. It was difficult to identify these lesions only by texture parameters values. With the further development of gastric cancers, the texture parameters values demonstrated great differences between gastric normal and cancer specimens and it was relatively easy to distinguish between gastric normal regions and gastric cancer regions by their texture parameters values.

### Results of PCA of the texture parameters based on GLCM

The new component can be expressed as F_i_ (i = 1,2,3…). When the CCR of F_1_ and F_2_ of ROIs of a gastric specimen is greater than 80%, F_1_ and F_2_ are used as principal components. Results of PCA based on GLCM are shown in Table 1.

**Table 1:** Results of the PCA of texture parameters based on GLCM (Mean ± Standard Deviation)

**PCA**	**Normal**	**3-days**	**5-days**	**7-days**	**9-days**	**11-days**
F_1_	7.01 ± 0.09	7.52 ± 0.09	7.13 ± 0.09	4.76 ± 0.07	7.29 ± 0.09	7.01 ± 0.09
F_2_	1.68 ± 0.04	0.85 ± 0.03	0.99 ± 0.03	2.51 ± 0.05	0.88 ± 0.03	1.00 ± 0.03
F_1_ + F_2_	8.69 ± 0.13	8.37 ± 0.12	8.12 ± 0.12	7.27 ± 0.12	8.17 ± 0.12	8.01 ± 0.12
CCR	96.51%	92.99%	90.26%	80.82%	90.79%	89.02%

The sum of the two principal component values of gastric normal regions is always greater than the sum of the two principal component values of gastric cancer regions, namely gastric normal regions (F_1_ + F_2_) > gastric cancer regions (F_1_ + F_2_). It is concluded that PCA of gastric normal regions is (F_1_ + F_2_) > 8.5 and PCA of gastric cancer regions is (F_1_ + F_2_) < 8.5 from Table 1. So this is very helpful for the auxiliary detection of gastric cancers.

### Results of gastric cancers classification using SVMs

**Figure 7:**
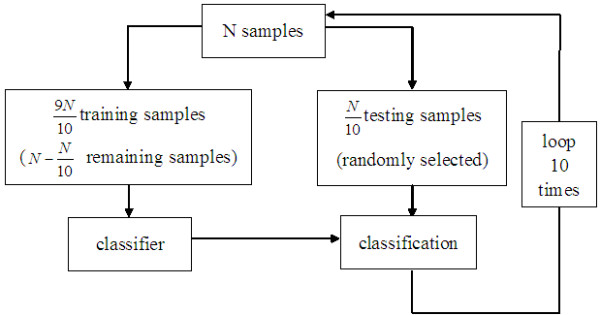
**The flow chart of 10-fold cross-validation using SVMs.**$\frac{N}{10}$ testing samples are randomly selected from all of N samples. $\frac{9N}{10}$ training samples are the remaining samples excluding testing samples. All of N samples are used as testing samples only once.

In this paper, we randomly selected 20 ROIs from each of XILPCI projective images of a normal and 3-, 5-, 7-, 9- or 11-day-old gastric specimens. The 9 GLCM texture parameters values were carried out on each ROI. So we built 120 samples and each sample included 9 characteristic parameters. $\frac{N}{10}$ (12 samples) samples were randomly selected as testing samples, and the other $\frac{9N}{10}$ (108 samples) samples are used as training samples. We analyzed the samples using SVM by 10-fold cross-validation, and our algorithm flow was shown in Figure 7. When linear kernel function was used, the result that the sum of 10 results was averaged was 83.3%. The SVMs result showed that different stages of gastric specimens can be classified using SVMs.

## Conclusions

In summary, we have applied XILPCI method to the imaging of gastric cancers in nude mice without the use of imaging agents. The contrast projective images showed that the development of gastric cancers may cause thickenings on gastric wall and coarseness on gastric textures. An XILPCI image of a gastric specimen can provide better contrast resolution than an X-ray traditional absorption image without the use of imaging agents. The image of phase contrast CT is also better than that of X-ray absorption CT. Moreover, texture analysis performed by using GLCM method illuminates that texture parameters values are monotonically increasing or decreasing trend with the growth of the gastric cancers. Through PCA method based on the GLCM texture parameters, it has been shown that the sum of principal component F_1_ and F_2_ can distinguish between gastric normal regions and gastric cancer regions. We also use the SVMs method for the first time to classify different stages of gastric specimens. Our work presented here shows that XILPCI has the potential to display stomachs with high anatomical accuracy.

## Discussions

The XILPCI is a new imaging method that differs from the X-ray traditional absorption imaging method, and the basis of XILPCI is X-ray phase variation. XILPCI method can realize micron-scale biological tissue without the use of imaging agents. In present, the XILPCI applications are primarily in the experimental stages. XILPCI can work by using ordinary micro-focus X-ray sources instead of synchrotron radiation sources. This advantage can let the new XILPCI method widely apply in the future without high cost. However, XILPCI has some limitations, which will need to be overcome if it is to have wider applications. The XILPCI device of SSRF has a small field of view. It takes us approximately 20 minutes to acquire images from the degree of 0 to 180. It needs around one hour to shoot for a larger specimen because the larger specimen needs shoot images into several segments. If XILPCI wants to take living specimens, the device must to be improved to shorten the time of the specimens’ imaging. We use a kind of currently very common filter back projective image algorithm, but the reconstruction image of XILPCI CT has circular artifacts. Here needs to further improve the reconstruction image algorithm to obtain better tomography images of XILPCI. There is a CCD camera with the resolution of 0.74 μm in BL13W1 of SSRF, thus XILPCI should be able to resolve even the smallest blood vessels. An additional advantage of XILPCI CT over other noninvasive methods is that there is no need for any specific probes or contrast agents. Contrast agents of barium will be prohibited to use if a patient is suspected of gastrointestinal perforation or complete obstruction. It is applicable to all cases without the use of imaging agents. These advantages should facilitate applications of this method not only to the imaging of gastric cancers but also to that of other pathological structures. In future studies, we will measure the blood vessels around gastric cancers.

Our results indicate that XILPCI provides a potential auxiliary way of early detection of gastric cancers. However, an extensive and systematic comparison between the XILPCI images and histopathology is needed to make these contrast mechanisms as diagnostic tools. Biopsy is a necessary and sufficient test and remains the gold standard for diagnosis of gastric cancers. Texture characteristics of these suspected cancer cases have the same texture characteristics reported in this paper, but other tests such as blood tests and biopsies are still needed. XILPCI requires further researches before its clinical applications. With further researches, XILPCI could become a valuable noninvasive method for future medical researches.

## Competing interests

The authors declare that they have no competing interests.

## Authors’ contributions

QT conceived of the study, and completed the experimental data, and worked on the algorithm design, and drafted the manuscript. SL contributed to suggestions throughout this topic. Both authors read and approved the final manuscript.

## Authors’ information

About the Author—Shuqian Luo, currently is a Full Professor, Biomedical Engineering Institute, Capital Medical University, Beijing, China, director of Medical Imaging Lab, IEEE Senior Member, project Leader: Multi-Modality Medical Image Registration, Brain Tissue Segmentation and Classification, 3D Digitalized Human Brain Atlas, Chinese Digital Human. He is editor of many Chinese journal and principle investigator of many projects, including the National High Technique Research and Development Plan (863 Plan), Project of National Natural Science Foundation. He won Henan Science and Technology Progress Second Place Award, Project of Multi-Functional ECG Analyzer, 1993, and Asian Ten major CT (Computed Tomography) Science Award, Project of Multi-Modality Medical Image Registration, 1999, and Beijing Traditional Chinese Medicine First Place Award, Project of Meridian Adjustment Diagnosis and Therapy System, 2001.3. Prof. Luo has published 150 papers and 6 books.

About the Author—Qiang Tao, currently is a lecturer, Biomedical Engineering Institute, Capital Medical University, Beijing, China. She has received the Doctor of Science degree from Biomedical Engineering Institute, Capital Medical University, Beijing, China in 2013. Her research interests are medical image processing and phase-contrast imaging.
